# Dance Tempo Estimation Using a Single Leg-Attached 3D Accelerometer

**DOI:** 10.3390/s21238066

**Published:** 2021-12-02

**Authors:** Sara Stančin, Sašo Tomažič

**Affiliations:** Faculty of Electrical Engineering, University of Ljubljana, 1000 Ljubljana, Slovenia; saso.tomazic@fe.uni-lj.si

**Keywords:** dance tempo, motion activation, motion analysis, inertial sensors, wearable devices, 3D accelerometer, solo jazz

## Abstract

We present a methodology that enables dance tempo estimation through the acquisition of 3D accelerometer signals using a single wearable inertial device positioned on the dancer’s leg. Our tempo estimation method is based on enhanced multiple resonators, implemented with comb feedback filters. To validate the methodology, we focus on the versatile solo jazz dance style. Including a variety of dance moves, with different leg activation patterns and rhythmical variations, solo jazz provides for a highly critical validation environment. We consider 15 different solo jazz dance moves, with different leg activation patterns, assembled in a sequence of 5 repetitions of each, giving 65 moves altogether. A professional and a recreational dancer performed this assembly in a controlled environment, following eight dancing tempos, dictated by a metronome, and ranging from 80 bpm to 220 bpm with 20 bpm increment steps. We show that with appropriate enhancements and using single leg signals, the comb filter bank provides for accurate dance tempo estimates for all moves and rhythmical variations considered. Dance tempo estimates for the overall assembles match strongly the dictated tempo—the difference being at most 1 bpm for all measurement instances is within the limits of the established beat onset stability of the used metronome. Results further show that this accuracy is achievable for shorter dancing excerpts, comprising four dance moves, corresponding to one music phrase, and as such enables real-time feedback. By providing for a dancer’s tempo quality and consistency assessment, the presented methodology has the potential of supporting the learning process, classifying individual level of experience, and assessing overall performance. It is extendable to other dance styles and sport motion in general where cyclical patterns occur.

## 1. Introduction

Dancing requires high levels of physical skills and body motion control. It has already been reported that dancers strongly benefit from various assistive technologies [1,2,3,4,5]. One of the essential dancing characteristics is the tempo of dancing, dictated by the accompanied music. Investigating and obtaining information regarding the tempo of a dancer’s motion execution can help beginners in the learning process and support dance move timing improvement. It can also help classify individual level of experience and assess overall performance. 

A number of dance tempo and timing estimation methods, capitalizing on the development and benefits of various sensing technologies, have been developed so far. Dancing Coach [5], for example, is a generic system designed to support the dancing practice by extracting dance steps using Kinect. In [6], another Kinect-based system is presented, enabling extraction and alignment evaluation of motion beats. Given a dance video clip as input, the system first extracts motion beats from the video and then measures how well the motion beats correlate with the music beats. The authors report an average F-score of about 80%. 

Keeping in mind that a dance assistive solution has to be practical and should not interfere with the dance performance itself, we present a robust methodology for dance tempo estimation that resides on a single 3D accelerometer sensor. Over the last years, it has been consistently demonstrated that inertial sensors are an efficient tool in the general research area of motion analysis [3,7,8,9,10,11,12]. Inertial sensors have become omnipresent and indispensable in various motion analysis applications. Their characteristic light weight, small size, low power consumption, portability, easiness of use, and low cost paved the way for their current presence in dance motion analysis. One of the first wireless systems with inertial sensors used for capturing dance gestures have been mounted on the toe and heel [11,12].

The problem of dance tempo estimation falls into the broader and well-studied research domain of fundamental frequency determination of quasi-periodic signals. The simplest approaches are based on the investigation of the time domain waveform and finding peaks or zero-crossings that are suitably apart, e.g., [3,5], and autocorrelation calculation, e.g., [13]. Since a periodic waveform is also characterised by regular grids of peaks in the spectral domain, common approaches also reside on calculating and analysing the spectral components, e.g., [14]. 

In a dance context in particular, estimating the step period by analysing the waveforms in the time domain has been reported in [3,5]. In [3], the authors explore how a smartphone accelerometer can be used to capture motion data while the user is using a prototype mobile app. By detecting the local maximums of the acceleration signal, a dancer’s dance tempo is estimated and compared to the reference tempo of the song. Saltate! [5], for example, is a wireless system that acquires data from force sensors mounted under the dancers’ feet, detects steps, and compares their timing to the timing of beats in the music playing. For detected mistakes, the system emphasises the beats acoustically to help dancers stay in sync with the music.

Our dance tempo estimation method avoids extracting steps in the time-domain. The problem of feature extraction in the time-domain is that it is prone to noise and fails for dancing styles that include other dance motion elements besides steps and are not represented by nearly periodic acceleration signals maximums. An example of such a dance style is solo jazz—a rhythmical and playful solo dance in which the dancer uses his movement to depict jazz music. Due to its versatility, involving moves with different leg motion and activation patterns, solo jazz fits perfectly into our tempo estimation setting.

To enable the dancer to follow the jazz song rhythmical structure, as a rule, a single solo jazz move is performed following eight music beats. The music beat is considered as the smallest time interval between two successive notes in the music rhythmic phrase. In general, dance motion executed following the music beats differs with respect to leg activation and motion elements. Leg activation patterns refer to changes in the dominantly executing leg. In general, such as while walking, dance moves can be executed moving the right and left leg alternately—after each step executed with one leg, the dominantly executing leg changes. If such is the pattern, a single leg step is executed every two music beats. In a vast number of dance styles, including solo jazz, more versatile leg activation patterns are also possible, allowing the dominantly executing leg to change every second or every fourth music beat. When compared to walking, we can imagine this as stepping twice (or four times) with one leg and then doing the same with the other leg. In addition, the dominant leg can be executing some other motion element, besides stepping, e.g., kicks, taps, and more or less subtle jumps. The variability of the leg activation patterns and motion elements, executed in between beats and consecutively assembled, brings forth a diversity of predefined authentic solo jazz dance moves. In general, the more experienced a dancer is, the more variability is present in her dancing. The most experienced dancers include idiosyncratic moves and syncopations to the authentic solo jazz repertoire.

The speed of execution represents the dance tempo and is directly related to the music tempo of the song to which the dancer is dancing. As a rule, solo jazz is danced to music with tempo anywhere between 120 and 250 beats per minute (bpm). A hobbyist dancer is usually the most comfortable dancing at intermediate tempos. The more experienced a dancer is, the more precise is her tempo, especially when dancing at tempos at the higher end of the emphasized range.

To estimate the dance tempo, we chose to implement a solution based on comb filters. The periodic magnitude response of the comb filter can be tuned to the fundamental frequency of dancing and its harmonics, enabling efficient dance tempo estimation. Avoiding spectrum calculation, the comb filter is widely employed because of its computational efficiency [15,16,17]. In addition, a comb filter method has already been shown reliable for estimating the tempo of dance music [17]. 

In dancing in general, due to inaccuracies in step execution, the signals are nearly periodic. In addition, rhythmical variability in solo jazz dance moves introduces significant additional changes of the frequency representation of the acquired signals and its fundamental frequency, exceeding the variability that can be accounted for by considering the quasi-periodic model.

Improvements and performance enhancements to the original comb filter enable us to accurately estimate the tempo of dancing even when the fundamental frequency of the acquired signals, due to rhythmical variations, changes significantly.

This article is organised as follows. In Section 2, we present the materials and methods implemented for dance tempo estimation. In Section 3, we present and discuss the experimental validation results. In Section 4, we summarise our findings and draw conclusions, implying further research directions. 

## 2. Materials and Methods

### 2.1. Data Acquisition

#### 2.1.1. Materials

We capture dance motion using an mbientlab MetaMotionR (MMR) wearable device [18], including a micro-electromechanical system (MEMS) 3D accelerometer. Aiming to provide easiness of use, with the smallest amount of sensing equipment, we rely on a single wearable unit. In such a setup, the optimal position for estimating the dance tempo would be the torso of the dancer (or above). However, by positioning the sensor on the dancer’s leg, we also provide for further possibilities of leg motion analysis. 

**Figure 1:**
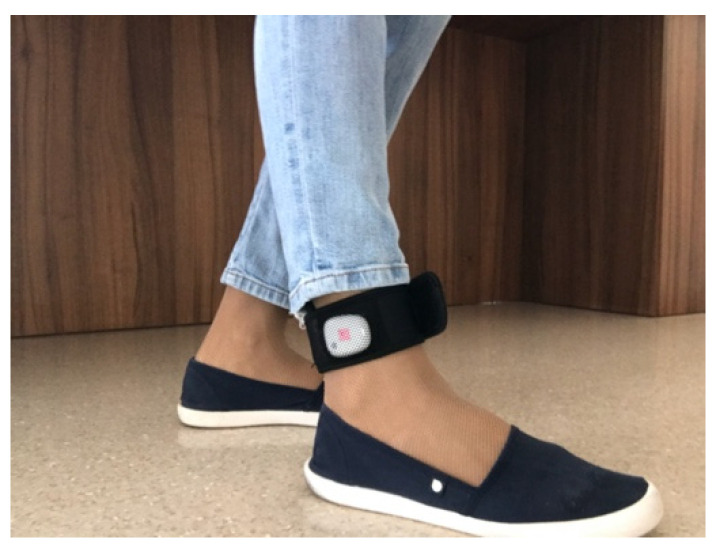
Dance motion capture. We use a single wearable device, including a micro-electromechanical system (MEMS) 3D accelerometer sensor, attached just above the dancer’s right leg ankle. The microposition and orientation of the sensor are arbitrary.

The specific position of the device is just above the dancer’s right leg ankle, as illustrated in Figure 1. Naturally, to capture dance motion mainly executed with the left leg, an additional sensor, attached to the left leg ankle, would also have to be used. Considering the relatively low probability of such moves, we choose not to focus on these situations, giving our attention to the simpler, single inertial device solution.

The microposition and orientation of the device are arbitrary as they proved not to affect the results. We set the sampling frequency to 200 Hz, which proved to be sufficient for the problem at hand using empirical evidence. In addition, a software metronome was used to dictate the dance tempo and simulate a steady quarter note music tempo.

#### 2.1.2. Measurements

Two female dancers participated in the study—one professional and one recreational with 3 years of solo jazz dancing experience. The same measurement routine was performed for each participant, during different measurement sessions.

Following the obtained instructions, the participant attached the wearable sensing device to her right leg ankle, voluntarily setting the device’s microposition and orientation. The participant then repeated a specified assemble of 65 consecutive dance moves for 8 different dance tempos, ranging from 80 bpm to 220 bpm with 20 bpm increment steps. A dance tempo of 240 bpm proved to be too high for the recreational dancer to execute with comfort and ease and was so not considered. It is necessary to note that the two lowest tempos considered, i.e., 80 and 100 bpm, are, in general, too low for the solo jazz dance context. However, their inclusion in the study enables a more robust validation and generalization to other dance styles and motion contexts.

The specified assemble included 5 repetitions of 15 authentic solo jazz dance moves. The following moves were included: (1) Tackie Annie; (2) Fall of the log; (3) Kicks; (4) Half break; (5) Struttin’; (6) Savoy kick; (7) 20′s Charleston; (8) Knee slaps; (9) Fishtails; (10) Apple Jacks; (11) Boogie back; (12) Boogie forward; (13) Crazy leg; (14) Cross step; and (15) Shorty George. For an informative overview of how these moves are performed, the reader is guided to various sources available online, e.g., [19]. Each of the 65 moves was performed on an 8-beat basis.

Each move, as will be debated in more detail in the following subsection, has a unique pattern of leg motion elements and activation, leading to rhythmical variability. Due to this variability, while all moves are performed on an 8-beat basis, dictated by a consistent tempo, their fundamental frequency can be different, having a significant effect on the frequency representation of the acquired signals.

Capturing the execution of the specified assemble for each dance tempo gave us a testing sequence of 3D acceleration signals. For the 8 considered dance tempos and 2 participants, we obtained 16 testing sequences in total. 

Both dancers gave oral consent in participating in the study and reported dancing with comfort to all considered tempos. All measurements were supplemented with video recordings and labels.

### 2.2. Signal Processing

#### 2.2.1. Signal Pre-Processing

Since the device itself does not provide outputs at exactly equidistant time samples, we interpolated and decimated the acquired signals as necessary, considering the associated measurement timestamp values, to provide for uniform sampling at exactly 200 Hz. Following the calibration procedure presented in [20], we compensated the signals for sensor inaccuracies. To remove motion artefacts and noise, we applied a low-pass filter with a cut-off frequency *f_co_* = 50 Hz and finally performed downsampling to *f_s_* = 100 Hz, obtaining 3D acceleration at equidistant time samples, *T* = 1/*f_s_* = 0.01 s. 

We normalized each of the three acceleration components to have a zero mean and unit standard deviation. We denoted the three obtained components’ signals, each of length *N* and given in the device-intrinsic coordinate system, as *a_x_*, *a_y_*, and *a_z_*. The specific orientation of the axes in a reference coordinate system is irrelevant.

#### 2.2.2. Dance Tempo Estimation

With the assumption that dance tempo is roughly constant throughout the analysed excerpt and that dance steps are, in general, nearly equidistant, with occasional inclusion of rhythmical improvisations and syncopations, estimating the dance tempo *υ* essentially refers to estimating the smallest inter step-onset interval *T_υ_*. In the most simplified scenario, for each music beat, one dance step is executed. Measuring *T_υ_* in seconds and *υ* in beats per minute, we can write

(1) $$T_{\upsilon }=\frac{1}{f_{\upsilon }}=\frac{60}{\upsilon }.$$

In Equation (1), *f_υ_* denotes the fundamental dance frequency, related to step execution and measured in Hz. Depending on step styling, a number of *f_υ_* harmonics are present, to a varying extent. In the given context, we expect *f_υ_* to be dominant over its harmonics.

In general, such as while walking, dance moves are executed moving the right and left leg alternately—after each step executed with one leg, the dominantly executing leg changes. Since single leg steps are executed every two beats, in our single-leg sensor setup, the smallest inter step-onset interval for a single leg is *T_step_* = 2*T_υ_*, introducing *f_step_* = *f_υ_*/2 as the fundamental frequency. We can write

(2) $$T_{step}=\frac{1}{f_{step}}=\frac{T_{\upsilon }}{2}=\frac{120}{\upsilon }.$$

In Equation (2), *f_step_* denotes the single leg step fundamental frequency, measured in Hz. In addition, for each *h* component of the original signal, *hf_υ_* ± *f_step_* are now present to a various extent. In such a simplified scenario, by detecting steps, by means of feature extraction in the time-domain, dance tempo can be estimated. 

Further on, in a more realistic scenario, besides changing the dominantly executing leg on every beat, dance moves can be executed with other leg activation patterns. In particular, besides on every beat, in solo jazz the dominantly executing leg can change on every second or every fourth beat. Every such variation introduces new components to the spectral content of the signals. When leg change occurs every two beats, the spectral content is enriched for components *hf_step_* ± *f_step_/2*, making *f_step_/2* the fundamental frequency. Likewise, when a leg change occurs every four beats, the spectral content is enriched for components *hf_step_* ± *f_step_/4*, making *f_step_/4* the fundamental spectral component. More complex rhythmical variations, such that in one move, different leg activation patterns mix, are also possible. 

**Figure 2:**
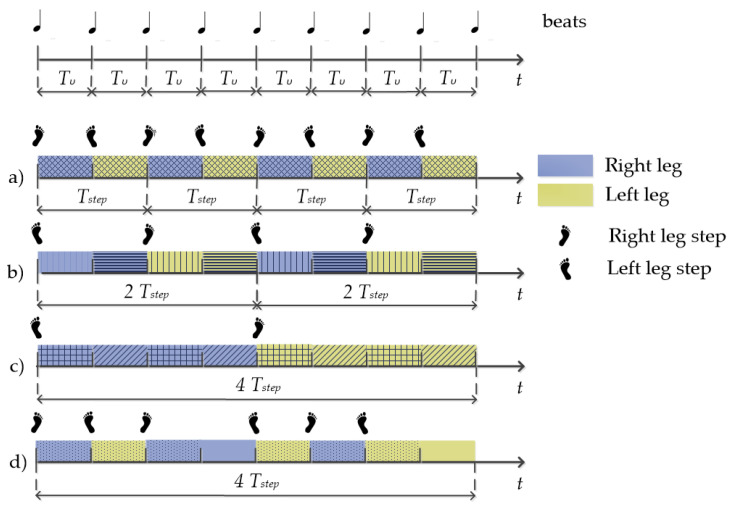
Leg activation patterns in solo jazz dancing. The first row illustrates the music beats that the dancer follows and define the dance tempo. The remaining rows illustrate different examples of leg activation patterns. Periods in which one leg is dominant are color-separated from periods of the other leg’s domination. Different hatch fills emphasize the difference in the executed motion elements. Examples (**a**–**c**) illustrate patterns of leg change on every beat, every two beats, and every four beats, respectively, while example (**d**) illustrates a more complex leg activation pattern.

Simplified and schematised leg activation patterns, characteristic for solo jazz, are illustrated in Figure 2. Example (a) illustrates the leg-change-on-every-beat pattern. Examples of solo jazz moves that follow such a pattern are (10) Apple Jacks; (11) Boggie back; (13) Crazy legs; and (15) Shorty George. Example (b) illustrates the leg-change-on-every-two-beats pattern, typical for (1) Tackie Annie; (3) Kicks; (9) Fish tails; and (12) Boggie forward solo jazz moves. Example (c) illustrates the leg-change-on-every-four-beats pattern, typical for (6) Savoy kick; (7) 20s Charleston; and (8) Knee slaps moves. Some solo jazz dance moves have a more complex pattern, where the leg change occurs either on a beat or on a two-beat basis, e.g., (2) Fall of the log; (4) Half break; (5) Struttin’; and (14) Cross step. Example (d) depicts the execution of one (14) Cross step.

This brings us to the conclusion that the frequency spectrum of the acquired signals varies, depending on the included moves. When danced at tempo υ, in a single sensor measurement scenario, the fundamental frequency of the acquired signals can be *f_step_*, *f_step_*/2, or *f_step_*/4. Therefore, we can conclude that *f_step_* is the lowest common component, regardless of the performed assemble. Its intensity relative to other components varies. For longer and diverse assembles of moves, we can expect *f_step_* to be the maximum frequency component. However, this is far from a straightforward conclusion for short dancing excerpts or assembles with a repeating pattern of a small number of moves. For the considered *υ* range 80–220 bpm, *f_step_* is between 0.67 and 1.83 Hz.

To estimate *f_step_*, we rely on multiple resonators implemented with IIR comb feedback filters. Opting for the comb feedback filter is reasoned with the filter’s periodic frequency response—a feedback comb filter resonates at *f_comb_* and all its harmonics. Using dance acceleration signals as filter inputs, a comb filter produces the highest energy of the output when the resonating frequencies match *f_step_* and its harmonics. By analysing and comparing outputs of multiple filters, each with a different resonating frequency, we can estimate the most likely value of the step frequency and therefore the dance tempo. Such an approach has already been proven to enable estimation of a song’s quarter note tempo [17], which is essentially dictating the dancer’s dance tempo.

In particular, to accommodate the considered dance tempo range, we implement a filter bank consisting of 151 comb feedback filters with the filter’s delay *k* ranging from *k_min_* = 50 to *k_max_* = 200 samples. Each *k* identifies the first peak of the magnitude response of the filter:

(3) $$f_{comb}=f_{s}/k.$$

For *f_s_* = 100 Hz and the set *k* range, *f_comb_* is increasing non-uniformly from 0.5 to 2.00 Hz. This range translates to, considering *f_step_* instead of *f_comb_* in Equation (2), the extended dance tempo *υ* range of interest, 60–240 bpm (with ever larger increment steps: 60.00, 60.30, 60.61, …, 235.29, 240.00 bpm). To note, the number of filters in the bank can be adjusted with respect to the plausible dance tempo range. Lowering the number of filters considered, by considering a limited tempo range can be particularly beneficial when optimizing run-time execution.

We use the normalized 3D acceleration signals as filter bank inputs. For each acceleration component signal, *a_x_*, *a_y_*, and *a_z_*, and for each delay *k*, the respective filter outputs *a_xf k_*, *a_yf k_*, and *a_zf k_* for each time sample 1 ≤ *n* ≤ *N* is calculated according to the following implementation equations:

(4) $$\begin{array}{l} a_{xf\;k}\left[n\right]=a_{x}\left[n\right]+\alpha a_{xf\;k}\left[n-k\right]\\ a_{yf\;k}\left[n\right]=a_{y}\left[n\right]+\alpha a_{yf\;k}\left[n-k\right]\\ a_{zf\;k}\left[n\right]=a_{z}\left[n\right]+\alpha a_{zf\;k}\left[n-k\right]. \end{array}$$

We assign a fixed gain α in Equation (4) to each filter in the bank. Overall best results were obtained for α=0.7.

For each filter in the bank, we compute the energy of the output cumulatively across all three acceleration dimensions, for each delay *k* according to

(5) $$e\left[k\right]=\sum_{n=0}^{N}{\left(a_{xf\;k}\left[n\right]\right)}^{2}+\sum_{n=0}^{N}{\left(a_{yf\;k}\left[n\right]\right)}^{2}+\sum_{n=0}^{N}{\left(a_{zf\;k}\left[n\right]\right)}^{2}.$$

Considering Equations (3) and (5) and after resampling, we obtain *e*[*f_comb_*], i.e., the energy of the output for equidistant values *f_comb_*, corresponding to 60, 61, 62, …, 239, and 240 bpm dance tempos. 

For a particular filter in the filter bank, tuned to the tempo of the analysed dancing the most, the energy of the output should be the highest. Considering this, we find the frequency *f_max_* of the output energy maximum:

(6) $$e\left[f_{max}\right]=\max\left\{e\left[f_{comb}\right]\right\}.$$

For longer dance assembles of various moves, *f_step_* is the dominant frequency component and should match the frequency of the filter with the maximum energy output. By setting

(7) $$f_{step}=f_{max}$$

and inserting in Equation (2), the dance tempo estimate follows:

(8) $$\upsilon_{est}=120f_{max}.$$

Note that the frequency range of the fundamental frequencies and higher harmonics for different *υ* in the considered 60–240 bpm range overlap. In addition, and particularly in the solo jazz dance context, for dance moves with leg change occurring every two beats, the comb filter tends to resonate stronger for *f_step_*/2 than for *f_step_*. If *f_step_*/2 falls into the considered range for which *e*[*f_comb_*] is calculated and the dance step frequencies are plausible, considering Equation (8) leads to an underestimate of the dance tempo. Precisely, for moves with *f_step_*/2 as the fundamental frequency, performed at *υ* and those with *f_step_*/4 as the fundamental frequency, performed at 2*υ*, the resulting comb filter responses are very much alike and it is not possible to uniformly estimate *f_step_* for both such cases, using Equations (4)–(8) only. Further on, for solo jazz dance moves with a leg change occurring every four beats, the comb filters tend to resonate stronger for *f_step_*/4 than for *f_step_*, leading again to an underestimate of the dance tempo if *f_step_*/4 falls into the considered comb filter frequency range. 

To account for the aforementioned particularities, we perform verification of the result of Equation (8) and eventually its correction. We do this by investigating the relative intensity of the fundamental dance frequency *f_υ_*, resulting from Equations (2) and (7), i.e., 2*f_max_*, to its harmonics. We expect *f_υ_* to be dominant.

First, for all *f_max_* multiples that fall into the considered frequency range, we check the significance of the associated value of the energy vector, relative to the significance of *e* [*f_max_*], by considering the difference between its value and the minimum value in its neighbourhood, according to

(9) $$\max(e\left[f_{comb}\right])-\min(e\left[f_{comb}\right])<s\cdot \left(e\left[mf_{\max}\right]-\min(e\left[mf_{\max}-\Delta f:mf_{\max}+\Delta f\right])\right).$$

In Equation (9), *m* determines the multiple (and can, for the considered frequency range of the comb filter and dancing tempos, be 2 or 4), *s* is the scaling factor set to 0.25, determined empirically as the best suit, and Δf is set to 0.15 Hz, making the neighbourhood width equal to 0.3 Hz, or a fifth of the entire *f_comb_* range. If Equation (9) holds for any multiple *m*, we consider the multiple that is closest to the upper limit of *f_comb_* and denoted with *Mf_max_*, as the new *f_step_* candidate, leading to a final *Mυ_est_* dance tempo estimate. We make the final decision between *υ_est_* and *Mυ_est_* by checking the associated fundamental dance frequency components’, i.e., *2f_max_* and *2Mf_max_*, relative dominance. We applied two IIR two poles resonators, one for each of the candidate frequencies, 2*f_max_* and *2Mf_max_*, to each component of the analysed acceleration signals:

(10) $$\begin{array}{l} a_{xr\;k}\left[n\right]=(1-\lambda )\sqrt{1+\lambda^{2}+2\lambda (1-2\cos^{2}(2\pi \frac{f_{c}}{f_{s}}))}\;a_{x}\left[n\right]+2\lambda \cos(2\pi \frac{f_{c}}{f_{s}})\;a_{xr\;k}\left[n-1\right]-\lambda^{2}a_{xr\;k}\left[n-2\right]\\ a_{yr\;k}\left[n\right]=(1-\lambda )\sqrt{1+\lambda^{2}+2\lambda (1-2\cos^{2}(2\pi \frac{f_{c}}{f_{s}}))}\;a_{y}\left[n\right]+2\lambda \cos(2\pi \frac{f_{c}}{f_{s}})\;a_{yr\;k}\left[n-1\right]-\lambda^{2}a_{yr\;k}\left[n-2\right]\\ a_{zr\;k}\left[n\right]=(1-\lambda )\sqrt{1+\lambda^{2}+2\lambda (1-2\cos^{2}(2\pi \frac{f_{c}}{f_{s}}))}\;a_{z}\left[n\right]+2\lambda \cos(2\pi \frac{f_{c}}{f_{s}})\;a_{zr\;k}\left[n-1\right]-\lambda^{2}a_{zr\;k}\left[n-2\right],\\ \mathrm{where}\;f_{c}=\left[2f_{\max\;}2Mf_{\max}\right]\;\mathrm{and}\;\lambda =0.995. \end{array}$$

We then calculate the energy output, by inserting the obtained filtered acceleration components Equation (10) instead of $a_{xf\;k},\;a_{yf\;k},$ and $a_{zf\;k}$ into Equation (5), for both candidate frequencies. We denote the obtained energy outputs for *2f_max_* and *2Mf_max_* with *e_r_*[*2f_max_*] and *e_r_*[2*Mf_max_*], respectively. If

(11) $$e_{r}\left[2Mf_{max}\right]>s_{r}e_{r}\left[2f_{max}\right],$$

where *s_r_* is the scaling factor again set to 0.25, determined empirically as the best suit, we correct *f_step_* by setting

(12) $$f_{step}=Mf_{\max}.$$

By inserting Equation (12) into Equation (2), the corrected dance tempo estimate follows:

(13) $$\upsilon_{est}=120Mf_{\max}.$$

### 2.3. Validation

We perform two sets of tests for methodology validation. For the first, we calculate the overall dance tempo, using the entire testing sequences as inputs. For each of the 16 testing sequences, we calculate the output energy according to Equations (4) and (5), find the location of its maximum and with respect to the conditions in Equations (9)–(11), and use either Equation (8) or Equation (13) as the dance tempo estimate. We calculate the absolute difference between the estimate and the dictated tempo as the final measure of result validation.

The presented methodology is computationally reasonably light. With the aim of establishing whether dance tempo can be accurately monitored and assessed in real time, for the second set of tests we calculate the dance tempo using short excerpts of the testing sequences as inputs. In particular, we consider the shortest excerpt length to be equal to the duration of one dance move, performed at the slowest tempo considered. We further assume that the tempo of dancing remains constant at least for a set of four dancing moves, representing one music phrase. At the slowest tempo considered, i.e., 80 bpm, a single move and an assemble of four consecutive dance moves are executed in 6 and 24 s, respectively. Under these assumptions, we set the excerpt length Δ, for which we perform tests, to be in the 6–24 s range, with 2 s increment steps. For *f_s_* = 100 Hz, these values translate to 600–2400 samples.

For each of the 16 testing sequences, we extract all subsequences of a specific length Δ and use them as short excerpts inputs for dance tempo estimation. Since our sequences are on average 30 × 10^3^ samples long, we obtained, on average, slightly under 30 × 10^3^ testing excerpts for each dance tempo and approximately 480 × 10^3^ excerpts altogether for all 16 sequences.

For each excerpt, we can calculate the output energy according to Equations (4)–(13). However, in order to reduce the computational complexity and accommodate for real-time processing, we assume that, for short excerpts, a reference dance tempo *υ_ref_* is known in advance. The reference tempo can be given either by the known tempo of the song or the target dance tempo. Considering that the dance tempo does not change abruptly and significantly, the reference tempo can also be estimated by analysing previous, longer excerpts of assembled moves. If shorter dance excerpts are to be analysed offline, the already established overall dance tempo can also be used.

Under the assumption of a known reference dance tempo, we can apply Equations (4)–(6) for a limited range of comb filter delays and use Equation (8) as the final dance tempo estimate. In particular, we set the limited range of the filters’ delays to be in between $k_{ref\;\min}=120\frac{f_{s}}{\upsilon_{ref}+\Delta \upsilon }\;\mathrm{and}\;k_{ref\;max}=120\frac{f_{s}}{\upsilon_{ref}-\Delta \upsilon },\mathrm{where}\;\Delta \upsilon$ is set to 15 bpm. The limited dance tempo estimation range is so 30 bpm, representing a third of the initial range and is sufficiently narrow to extract a single peak of the energy vector.

The aforementioned analysis in equivalent to establishing the local maximums of the energy output Equation (5) and using the frequency of the peak closest to the reference dance step frequency as the final estimate, without considering the entire initial *k* range.

As a final measure of result validation, we calculated the average absolute difference between the estimates and the reference tempo for all excerpts of a particular length and for a particular tempo, i.e., the testing sequence.

## 3. Results and Discussion

### 3.1. Overall Dance Tempo Estimation

**Figure 3:**
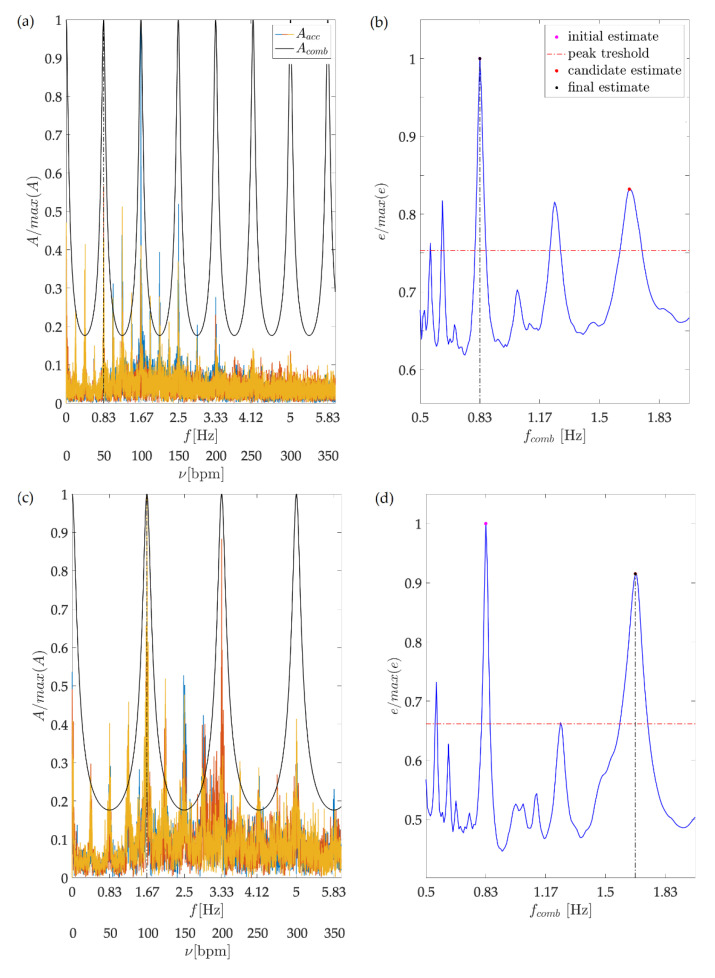
Overall dance tempo estimation. The first and second row refer to the recreational dancer’s 100 and 200 bpm testing sequences, respectively. Images (**a**,**c**) depict in colour the 3D acceleration amplitude frequency spectrum. For both examples, the spectrum has a clearly distinguishable peak at the respective step frequency, (**a**) 0.83 Hz for the 100 bpm tempo and (**c**) 1.67 Hz for the 200 bpm tempo). Depicted in black colour is the magnitude response of the tuned comb filter. Both plots are normalized to fit into to [0,1] range. Images (**b**,**d**) present the output energy, calculated for the entire range of comb filters frequencies together with step frequency estimates.

Figure 3 illustrates dance tempo estimation, performed for two testing sequence as presented in Section 2. For illustration purposes, we chose the testing sequences performed by the recreational dancer dancing at 100 (first row) and 200 bpm (second row), for which the single leg step frequencies are *f_step_* = 0.83 Hz and 1.67 Hz, respectively. The left column depicts the amplitude frequency spectrums of the analysed sequences, along with the tuned comb filter’s magnitude response. All plots are normalized to fit into the [0,1] range.

For both analysed sequences, we can observe a spectral maximum at the respective *f_step_*, representing half of the dance tempo. Besides this frequency, various other components are also distinguishable, including *f_step_*/2, *f_step_*/4. The presented frequency representation reflects the variability of solo jazz moves and leg activation patterns and is aligned with the reasoning and expectations presented in the previous section.

The right column presents the output energy, calculated for the entire *f_comb_* range. For both sequences, the maximum energy output is obtained for *f_max_* = 0.83 Hz. Considering *f_max_* as the step frequency would result in a dance tempo estimate of 100 bpm. We can further observe that for both sequences, the energy output has a significant peak at 2*f_max_*, giving the possibility of a 200 bpm estimate. From the presented signal frequency content for the first analysed sequence, it is visible that the component 100 bpm is dominant over the 200 bpm component. The resonator-based analysis performed according to Equations (9)–(11) confirms this and discards 2*f_max_* as the step fundamental frequency.

For the second sequence, it is visible that the 100 bpm component is not dominant over the component 200 bpm. The analysis corrects the initial estimate and sets 2*f_max_* as the step frequency. In both cases, correct estimates are obtained, matching perfectly the tempo dictated by the metronome.

Complete results, obtained for all 16 testing sequences, are presented in Table 1. For all but one, the estimated tempo matches perfectly the tempo dictated by the metronome. The only sequence for which the estimated tempo differs from the tempo dictated by the metronome is the professional’s 120 bpm sequence. In an absolute sense, this difference is 1 bpm, which is in the range of the established beat onset stability of the used metronome.

These results confirm that (1) both dancers are dancing with an accurate tempo overall; and (2) the presented methodology provides for accurate dance tempo estimation.

### 3.2. Dance Tempo Estimation for Short Excerpts

**Figure 4:**
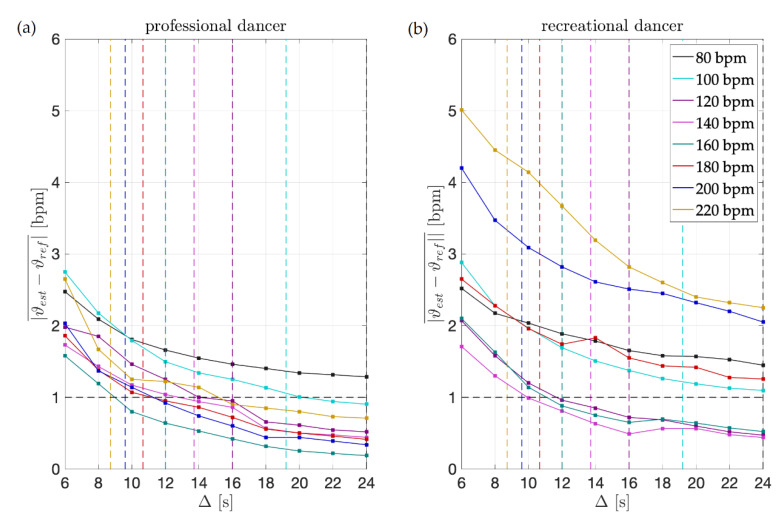
Dance tempo estimation results for short dance excerpts. Each vertical, colored line additionally illustrates the duration of four consecutive dance moves, for each dance tempo respectively. For (**a**) the professional dancer for excerpts of such duration, the results are at the level of 1 bpm accuracy for all tempos. For (**b**) the recreational dancer, this holds only for the intermediate tempos, i.e., 100, 120, 140, and 160 bpm.

The results obtained for short excerpts of sequences are presented in Figure 4. For all sequences, the overall tempo results, as presented in Table 1, are used as the reference tempo.

The first column presents the results obtained for the professional dancer’s excerpts. For the shortest excerpts’ duration considered, i.e., 6 s, the mean absolute difference between the estimates and the reference tempo is between 1.5 and 3 bpm. We can speculate that besides being a consequence of the methodology itself, in particular due to the inherent time-frequency indetermination, such levels of deviations might also be a consequence of the dancer’s execution. However, at the levels of duration of one music phrases, i.e., four dance moves, which is additionally depicted in the image for each tempo, the difference is below 1.5 bpm and as such is in the range of the overall tempo estimates accuracy and metronome beat onset stability.

Again, the results confirm two things: at the level of duration of four dance moves (1) the professional dancer is dancing with an accurate tempo; and (2) the presented methodology provides for accurate dance tempo estimation. Enhancements to the original comb filter performed enable us to accurately estimate the tempo of dancing even when analysing short excerpts where the reasonable length of the excerpts corresponds to four dance moves.

The second column presents the results for the recreational dancer’s excerpts. Instantly observable are considerable deteriorations in results, across all tempos and excerpt durations, when compared to the results obtained for the professional dancer. For the shortest excerpts, the mean absolute difference between the estimates and the reference dance tempo is as high as 5 bpm.

Further on, for the recreational dancer’s excerpts comprising four moves, the results are at the accuracy level of the overall dance tempo estimates only for tempos up to 160 bpm. This brings us to the conclusion that while dancing with an accurate tempo overall, when dancing at higher tempos, the recreational dancer does not achieve the same levels of dance tempo accuracy as the professional dancer and that the presented methodology can help in assessing the quality of performance, online and on a short excerpt basis.

To finally note, for both, the professional and the recreational dancer, the largest errors for excerpts comprising four moves are obtained for the slowest and fastest tempos considered (80 bpm for both dancers and 220 bpm for the professional and 200 and 220 bpm for the recreational), confirming that both dancers are more precise when dancing at intermediate tempos.

## 4. Conclusions

Using a single 3D accelerometer positioned on the dancer’s leg and entailing enhanced multiple resonators implemented with IIR comb feedback filters has proven to provide for accurate dance tempo estimation. Enhancements to the original comb filter performed enable us to efficiently estimate the tempo of dancing despite the changes in the fundamental frequency of the acquired signals, due to rhythmical variations.

The results obtained show that when analysing assembles of various moves, performed at a constant tempo, for both the professional and the recreational dancer, the difference between the estimate and the dictated tempo is at most 1 bpm, and as such is in the range of the established beat onset stability of the used metronome.

Assuming that the tempo of dancing remains constant for an assemble of four consecutive dance moves, representing one musical phrase, it is reasonable to interpret real-time feedback as providing online dancing tempo estimates referring to the last four moves, approximately. The validation performed for excerpts of such a duration shows the result accuracy is at the same level as the accuracy of overall dance tempo estimates.

When comparing how well the dancers maintain a steady dance tempo throughout the dance sessions, the professional dancer clearly outperforms the recreational one, giving the technology an applicative value of assessing the quality of performance in real time.

Levels of accuracy obtained open new topics for further research. Besides assessing the dancer’s tempo quality and consistency, dance tempo estimates can support detecting dance steps and other motion patterns. Relating these to the music beats, off-beat steps can be detected, and the dancer’s timing quality can be analysed. By analysing the rhythmical patterns, the dancer’s response to music can be monitored. All of this together can provide for a technology that would support estimating the crucial connection between dancing and music. Additionally, using dance tempo estimates, dancing sequences can be temporally normalized and segmented. Reference motion patterns can be created. By comparing the analysed motion to the reference patterns, quality of execution can be established.

Residing on a single inertial sensing device and avoiding using video cameras or IR imaging sensors, the presented solution is highly suitable for the abovementioned challenges. Due to its computational efficiency and portability, it can be in a variety of dancing situations—when the dancer is dancing alone, in the crowd, or in front of an audience.

Further analysis is necessary to investigate the methodology performance in real-case dancing scenarios, with various levels of dancing improvisation and syncopation that would help investigate the individual style of the dancer and assess her levels of dancing vocabulary and creativity.

Finally, since some parameters of the analysis are related to the testing tempo range and the characteristics of solo jazz dancing, if the methodology is applied to other problems, these should be fine-tuned.

## Figures and Tables

**Table 1:** Overall dance tempo estimation results. For all but one sequence, the estimated tempo matches perfectly the tempo dictated by the metronome. For the remaining sequence, i.e., the professional’s 120 bpm sequence, the absolute difference is 1 bpm, which is in the range of metronome beat onset stability.

Metronome Tempo (bpm)	Estimated Tempo (bpm)		Absolute Tempo Difference (bpm)
	Professional Dancer	Recreational Dancer	
80	80	80	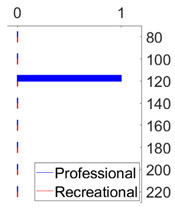
100	100	100	
120	121	120	
140	140	140	
160	160	160	
180	180	180	
200	200	200	
220	220	220	

## Data Availability

The data presented in this study are available on request from the corresponding author.
